# An Electron Spin Resonance Study Comparing Nanometer–Nanosecond Dynamics in Diblock Copolymers and Their Poly(methyl Methacrylate) Binary Blends

**DOI:** 10.3390/polym15204195

**Published:** 2023-10-23

**Authors:** Laura Andreozzi, Elisa Martinelli

**Affiliations:** 1 Dipartimento di Fisica, Università di Pisa, Largo Pontecorvo 3, 56127 Pisa, Italy; 2 Istituto per i Processi Chimico-Fisici-Consiglio Nazionale delle Ricerche (IPCF-CNR), Via G. Moruzzi 1, 56124 Pisa, Italy; 3 CISUP, Centro per l’Integrazione della Strumentazione dell’Università di Pisa, Lungarno Pacinotti 43/44, 56126 Pisa, Italy; elisa.martinelli@unipi.it; 4 Dipartimento di Chimica e Chimica Industriale, Università di Pisa, Via G. Moruzzi 13, 56124 Pisa, Italy

**Keywords:** electron spin resonance, copolymers, polymer blends, PMMA, photoresponsive polymers, self-assembly, nanoscale relaxation, structural relaxation, scaling law

## Abstract

Block copolymers are a class of materials that are particularly interesting with respect to their capability to self-assemble in ordered structures. In this context, the coupling between environment and dynamics is particularly relevant given that movements at the molecular level influence various properties of macromolecules. Mixing the polymer with a second macromolecule appears to be an easy method for studying these relationships. In this work, we studied blends of poly(methyl methacrylate) (PMMA) and a block copolymer composed of PMMA as the first block and poly(3-methyl-4-[6-(methylacryloyloxy)-hexyloxy]-4′-pentyloxy azobenzene) as the second block. The relaxational properties of these blends were investigated via electron spin resonance (ESR) spectroscopy, which is sensitive to nanometric length scales. The results of the investigations on the blends were related to the dynamic behavior of the copolymers. At the nanoscale, the study revealed the presence of heterogeneities, with slow and fast dynamics available for molecular reorientation, which are further modulated by the ability of the block copolymers to form supramolecular structures. For blends, the heterogeneities at the nanoscale were still detected. However, it was observed that the presence of the PMMA as a major component of the blends modified their dynamic behavior.

## 1. Introduction

Block copolymers are an interesting class of single-component polymeric materials [1] that cannot undergo macrophase separation. However, the chemically incompatible blocks, which are covalently linked, lead to local microphase segregation at the nanoscale and self-assembly [2] (pp. 24–130). Their bulk organization can exhibit various morphologies [3,4,5] at nanoscopic length scales; the typical dimensions of the microdomains range from 5 to 50 nm [6], a span that encompasses those required for quite an extensive range of potential applications [7,8,9].

Research progress in materials chemistry has provided polymerization techniques [10,11,12], giving the opportunity to access a substantial variety of polymer architectures that range in complexity and have controllable multifunctions [7,8,9]. Block copolymers containing liquid crystalline (LC) moieties have attracted scientific and industrial attention because of the possibility of adding orientational ordering to the morphological characteristics of block copolymers [13]. Their potential applications span the fields of biology, photonics, nanotemplates, and nanofabrication processes [7,14,15,16,17,18,19].

A special class of LC block copolymers contains blocks with side-chain liquid crystalline (SCLC) units, namely, blocks with an amorphous backbone and mesogenic units attached to them as side chains, typically via an alkyl spacer [20]. A relevant unit that can be used as a mesogenic group in SCLC block copolymers is the azobenzene chromophore [21]. Indeed, because of their controllable properties in response to light [22,23], polymers embedding azobenzene moieties in their structure have been studied intensively as promising photonic materials. At the molecular level, over several spatial and temporal length scales, the reversible processes of photoorientation, photoselection, and photomodulation can be generated via repeated trans−cis−trans cycles of the azobenzene chromophore within a polymeric material. Then, the photoreaction is accompanied by a molecular reorientation and arrangement, which might be applicable, for example, to rewritable optical and holographic memories [24], optoelectronics [25], lithography [26], and nanomechanical actuators [27]. Soft mass migration and surface relief can also take place as a consequence of photoisomerization processes, providing new perspectives on the progress and evolution of devices in the field of micro/nanophotonics [28]. Moreover, the mechanisms of photoorientation, such as topological optical patterning and photomechanical actuation provided by azobenzene, allow light to control the molecular motion. These peculiarities open the possibility of applications in bio-interface tissue engineering and cell biology [29]. Furthermore, taking advantage of the photoresponsivity of their surfaces, azobenzene-functionalized side-chain polymers show reversible wettability and polarity. These features can open the field to surface technological applications [30].

Azo-based polymers have been demonstrated to be appropriate candidates for all-optical reversible information data storage at both micrometer and nanometer length scales [31].

To obtain increased information storage with respect to the films of azo-containing homopolymers, enhanced performance could be obtained using azobenzene block copolymers. In fact, microphase separation in nano-domains is a favorable factor for the performance of optically driven nano-modifications [32,33,34]. In fact, on the one hand, it leads to the long-term and high-reliability storage of light-induced nano-written information, and, on the other hand, it allows for both a decrease in the optical absorption of azobenzene units and an extension of possible information storage over the complete thickness of the sample [13,31]. In this respect, another interesting approach is provided by the blending procedure, which is a general method that is largely in use [35,36,37] for attaining a specified portfolio of physical properties without synthesizing specialized polymer systems. As an example, the preparation of blends of azobenzene block copolymers with a polymer that is structurally similar to one of the blocks enabled the enhancement of the dilution of the azobenzene moieties and the control of the microstructure of microsegregated copolymers. Their optical properties were investigated and characterized for optical storage purposes [38,39].

As a general feature, a recording medium suitable for all-optical reversible information data storage should exhibit a series of properties [24] such as optical quality, sensitivity, dynamic range, optical absorption, bit stability, homogeneity at a molecular level, and working temperature [40,41].

Accordingly, dynamics at the nanoscale are an important issue for the formation and stabilization of microstructures and relaxation processes, and its study appears to be a pivotal topic for understanding and characterizing materials.

In this respect, electron spin resonance (ESR) spectroscopy has proven to be important with respect to the study of dynamics and the local structure of simple [42,43] and complex [44,45,46,47,48,49] materials. In particular, ESR spectroscopy is highly sensitive to the segmental motion of polymer chains, which, in turn, can modulate the macroscopic properties and performances of polymers. In diamagnetic systems, ESR investigations are often carried out by dissolving very small quantities of spin probes, namely paramagnetic centers [50,51] that can select the length scale of the experiment using their size and shape. Among nitroxide spin probes, cholestane was employed profitably in the literature to assess molecular motion because its ESR spectrum is sensitive to the morphology and dynamics of the environment [41,50,51,52].

ESR spectroscopy has been profitably exploited to study different segmental dynamic responses at the nanoscales both in terms of time and length, allowing the obtainment of plenty of information. Using ESR, it was possible to detect heterogeneity and stability at the nanoscale of different molecular sites in polymers [42,53]. ESR investigations were able to reveal connections between nanoscale dynamics with chain dynamics or local relaxation [52,53,54]. They appeared to be associated with dynamic anomalies and signatures relative to the temperature-dependence behavior of molecular reorientation [55,56,57].

In the present publication, we study the blends of PMMA and two block copolymers comprising methyl methacrylate (MMA) as the first block and an azobenzene-based methacrylic (MA4) second block. We report the results of an ESR study on the blends, and this is also compared with a previous investigation carried out on the two neat block copolymers.

The focus is on dynamics at the nanoscale, which is a pivotal topic for the formation and stabilization of microstructures and relaxation processes in these materials. The aim is to unambiguously establish the distinct response of local dynamics as a consequence of matrix heterogeneity and changes in the environment. We provide evidence with respect to how different matrix heterogeneities, characterized by very different dynamic responses at the nanoscale, are generated by the different morphologies of materials.

## 2. Materials and Methods

### 2.1. Materials

**Figure 1:**
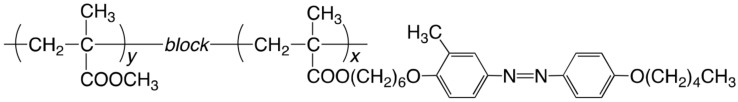
Diblock copolymer structure. B*x* (*x* = 20, 10 mol%, *y* = 100–*x*).

Diblock copolymers of the nematogenic 3-methyl-4-[6-(methylacryloyloxy)-hexyloxy]-4′-pentyloxy azobenzene MA4 monomer and methyl methacrylate MMA (Figure 1, Table 1) were prepared according to reported procedures [32,58].

The molar mass of the MA4 repeat unit is 468 g mol^–1^, and the length of its side chain is about 30 Å (evaluated with ChemSketch). The molar mass of the MMA repeat unit is 100 g mol^–1^, with a side-chain length of about 2.5 Å (evaluated with ChemSketch). The well-defined structure and low molar mass dispersion of the diblock copolymers were obtained via atom transfer radical polymerization (ATRP) according to a two-step procedure. In particular, during the first step, a PMMA macroinitiator with an average degree of polymerization of 200 and standard microstructure (60% syndiotactic, 35% atactic, and 5% isotactic) was synthesized (Table 1) and then used for the polymerization of MMA.

Blends of the copolymers with the same PMMA homopolymer, which was also used as macroinitiator, were prepared via the codissolution of the corresponding amounts of both components in dry dichloromethane. The final content of azobenzene units in the blends was 5% by weight.

Diblock copolymers are indicated as B*x*, where *x* denotes the 20% or 10% mole fraction of MA4 co-units in the copolymer (Figure 1). The blends containing 5% of the weight of MA4 co-units are indicated by adding a b5 prefix to the diblock copolymer names; thus, the blends are referred to as b5B10 and b5B20. In particular, b5B10 contains 15% of the weight of the B10 copolymer, while b5B20 has 9.5% of the B20 copolymer’s weight.

Size exclusion chromatography (SEC) experiments were performed on diblock copolymers [32]. Table 1 reports the obtained average molar masses and polydispersity. However, it was observed that a difference of 10% from the respective SEC values may affect the absolute values of the molar masses of these copolymer samples [62]. A Mettler Toledo DSC30 calorimeter was used to record DSC thermograms of the diblock copolymers, and these were registered at 10 K min^−1^ upon heating from about 225 K after having cooled from about 473 K at a rate of 20 K min^−1^.

A liquid crystalline phase was found in copolymers with a nematic-to-isotropic transition temperature, *T_NI_.* Moreover, they presented two glass transition temperatures, *T_g_*^PMMA^ and *T_g_*^PMA4^, as expected for microphase-separated block copolymers [63].

DSC studies on blends evidenced a loss in sensitivity with respect to the technique when the amount of a blended component was less than ~15% (see for example ref [64] and the references therein). Therefore, it is expected that only the glass transition temperature *T_g_*^PMMA^ may be macroscopically detected using the technique. A calculation of the expected T*_g_* values of the blends was carried out using the Utracki and Jukes equation [61]. A *T_g_* value of 386 K was obtained, confirming that the PMMA homopolymer mainly drives the thermal response of the blends.

Atomic force microscopy investigations in diblock copolymers showed the presence of an order-to-disorder transition (*T_ODT_*) (Table 1) [65], signaling that the copolymers were microphase-separated below *T_ODT_*. In binary blends of a homopolymer with a block copolymer, the phase behavior is controlled by the length of the homopolymer chain in comparison with the copolymer [2] (p. 332). Let us consider homopolymer A, with its number-average degree of polymerization being N_AH_, and the same A component in the copolymer, with its number-average degree of polymerization being N_AC_. In general, if N_AH_ ≈ N_AC_, a selective solubilization of the homopolymer in the A microdomains of the copolymer is present. However, there is a tendency of the homopolymer to remain in the middle of the A microdomains. As a consequence, the conformations of chain B, namely the ones of the other block of the copolymer, are not perturbed significantly. In all the present blends, the ratio between the number-average molecular weights (or the degrees of polymerization) of the homopolymer and the corresponding block in the copolymer is ρ = M_AH_/M_AC_ = N_AH_/N_AC_ = 1; thus, we expect the permanence of the microphase separation in the blends [2].

### 2.2. Rheological Characterization

An Anton Paar Physica MCR301 rheometer (plane–plate sensor system, 25 mm diameter) was used to characterize the viscoelastic response of diblock copolymers. The stability of the temperature of the samples was within 0.1 K (CTD450 temperature unit).

Oscillatory and creep-recovery experiments were carried out after having ensured their gap independence. The use of appropriate stress intensities allowed us to attain these experiments in the linear viscoelasticity regime [66]. Zero-shear viscosity *η* was evaluated by conducting creep and creep-recovery experiments [67] (pp. 419−464), [68]. Measurements of the complex shear modulus G* [66] were obtained using oscillatory isothermal dynamic experiments; the frequency range from 10^–3^ Hz to 24.4 Hz was usually employed. However, upon approaching *T_g_*, the lower frequency limit was widened down to 10^−4^ Hz.

Rheological material functions were investigated in the copolymers, from temperatures above *T_g_*^PMA4^ up to high temperatures in the flow region for B10 and B20.

No discontinuity was detected in the investigated temperature range [69], not even - across *T_NI_* and *T_ODT_*.

Viscosity temperature dependence was profitably fitted by the Vogel–Fulcher–Tamman (VFT) law [70]:

*η* (*T*) = *η* _∞_ exp [*T_b_*/*T* − *T*_0_] (1)

Vogel temperatures *T*_0_ and pseudoactivation temperatures *T_b_* are given in Table 2 [55].

However, a failure of the time–temperature superposition (TTS) principle [67] (pp. 486–491) [68] was observed when the reconstruction of a master curve for G* was attempted. This comes from the different structural and chemical arrangements of block copolymers.

In spite of the thermorheological complexity of copolymers, the superposition of isothermal frequency sweeps was obtained for the real *G*′ part [66] of the complex modulus G*. The possibility of collapsing to a single master curve the elastic component of G* could be ascribed to the different temperature dependencies of the monomeric friction coefficients of the blocks. This dependence affects the elastic properties to a lesser extent rather than the viscous ones [69].

In Table 2, the fitting parameters are also reported for the VFT dependence of the shift factor *a* (*T*) [71] of *G*′. In a thermorheologically simple polymer, the VTF parameters of viscosity and shift factor are found to be the same. The non-coincident values of the VFT parameters for *η* and *a* (*T*), as shown in Table 2 [55], confirm the presence of the different mechanisms that drive elastic and viscous behaviors in these diblock copolymers.

In Table 2, the rheological viscoelastic behavior data of a PMMA sample of similar molecular tacticity were taken from the literature [59].

Because of the low quantities of available samples, it was not possible to carry out rheology measurements over blends. The evaluations of the mixture viscosity could be roughly carried out by resorting to simple mixing rules. They add logarithms of the viscosity of components by weighing with their mole fraction or their mass fraction in the mixture. More sophisticated methods are also available in the literature [72,73].

### 2.3. ESR—Apparatus and Experimental Technique

A Bruker ER200D-SRC spectrometer (Bruker Corporation, Billerica, MA, USA) was used to perform ESR measurements. It operates in continuous wave and is equipped with an X band bridge (Bruker ER042-MRH) (Bruker Corporation, Billerica, MA, USA) and an NMR gaussmeter ER035M. A Bruker BVT100 (Bruker Corporation, Billerica, MA, USA) gas-flow unit controls the temperature with a nominal accuracy of ±0.1 K.

When ESR spectroscopy is carried out in liquids, the interest is devoted to fluctuating magnetic fields, which are randomly modulated by the motion of the lattice. They provide relaxation mechanisms to the spin system, which is embedded in the paramagnetic center. From the resultant lineshape detected in the ESR experiment, it is possible to infer quantitative details on the molecular motion and on the site where the paramagnetic center is located [74,75]. Due to the diamagnetic character of the copolymers and blends of this study, ESR measurements were carried out with the molecular probe technique in such a manner that the relaxation of the spin system of the guest molecule may provide information on the dynamics of the host matrix.

To this aim, the paramagnetic molecular tracer cholestane was dissolved in the polymeric samples. For the preparation of all samples, two chloroform solutions were mixed at room temperature, with each of them incorporating a determined quantity of copolymer or related blend and of molecular probe. The concentration of the sample solutions was about 10^−3^ with respect to the cholestane/repeat unit molar ratio. The samples were then evaporated to dryness and sealed in an ESR tube.

**Figure 2:**
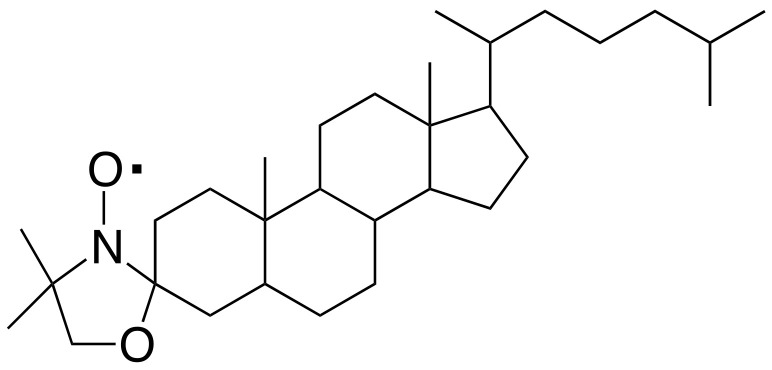
Structure of the cholestane molecular tracer.

The structure of the cholestane (3*β*-doxyl-5*α*-cholestane, 98%, Sigma-Aldrich, Merck KGaA, Darmstadt, Germany) nitroxide spin probe is shown in Figure 2. It was adopted because of good thermal stability and because anisotropic media are suitably probed by its shape and stiffness [41,55,57,76,77].

A prolate ellipsoid with semiaxes of about 0.99 nm and 0.29 nm can conveniently sketch the cholestane probe. The sensitivity of the ESR experiment at the nanometer scale [41,57] is selected by the size of the probe. The nanometer length scale is relevant for detecting heterogeneous morphology and the dynamics of cooperative processes in materials [78]. Moreover, the ESR lineshapes of the cholestane tracer are determined by examining rotational correlation times that fall in the nanosecond time scale of molecular motion, and this time scale is characteristic of the dynamics of complex systems [57,79,80].

The diffusion model under cylindrical symmetry may suitably describe cholestane’s rotational dynamics [81,82]. Accordingly, the rotation along the symmetry axis of the molecule is evaluated via spinning diffusion coefficient *D_||_*, and the rotation of the symmetry axis itself is accounted for via tumbling diffusion coefficient *D*_⊥_ [83,84]. With respect to cholestane, in all the investigated copolymers and blends, the *D_||_*/*D*_⊥_ ratio was close to 15. In order to compare the results of the rotational dynamics of this study with the ones reported in the literature [41,52,55] [50] (pp. 60–63), we discuss the reorientation of cholestane by examining the spinning correlation time, which is defined as *τ_||_* = 1/(6*D_||_*) [50] (pp. 60–63), [85,86].

The pure spin Hamiltonian, relevant to ESR experiments in liquids with a low concentration of nitroxide molecular tracers, includes Zeeman and hyperfine terms [74]. The principal component values of the Zeeman and hyperfine magnetic tensors of cholestane, expressed in the molecular frame [50,87], are reported in Table 3. In the table, for the sake of completeness, the principal component of the PMA4 homopolymer (Figure 1, *x* = 100 mol%) [41] and a PMMA (Figure 1, *y* = 100 mol%) homopolymer sample are also reported [56].

They were obtained via the numerical calculation of ESR lineshapes in the ultraslow motion regime [88], following a procedure detailed in ref. [81].

More details about the experimental procedure and ESR spectroscopy can be found elsewhere (see for example [54] and the references therein and the SI of [55]).

## 3. Results and Discussion

### 3.1. ESR Lineshapes

**Figure 3:**
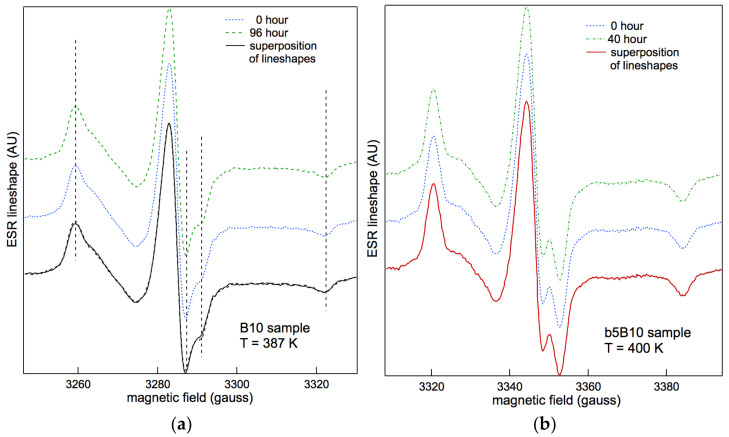
(**a**) ESR lineshape of B10 at the start and end of the annealing procedure at *T_a_* = 387 K [55]. (**b**) ESR lineshape of b5B10 at the start and end of the annealing procedure at *T_a_* = 400 K.

As a first step, ESR experiments were carried out to check for the presence of memory effects related to the thermal history of samples. Figure 3a shows the experimental ESR spectra for B10, which were recorded at an annealing temperature of *T_a_* = 387 K and set in the isotropic state of the LC copolymer. Lineshapes refer to the beginning and end of the annealing procedure. The lineshapes for the b5B10 blend, recorded at the start and end of the annealing procedure at *T_a_* = 400 K, are also shown in Figure 3b. None of the copolymers and blends in this study exhibited any thermal history dependence [41,55]; therefore, the study was carried out by the spectra at the selected temperatures without any thermal treatment.

These findings differ from what was reported in previous studies [41,52,55] with respect to liquid crystalline PMA4 random copolymers and homopolymers (*x* = 100 mol% in Figure 1), where memory effects affected the stability of the ESR lineshape, with a redistribution of the spin probe between different local environments. The small perturbation, induced in the liquid crystalline phase by the ordering process of mesogenic units, probably generates the site’s stability, which is detected in the blends and diblock copolymers [55]. These results seem to confirm the results of the literature [89] with respect to polymers that are similar to those of the present study, where it was found that the morphology of the microphase of block copolymers containing side-chain liquid crystalline units was not influenced by the liquid crystalline order.

The lineshapes in Figure 3, while referring to a sample with a relatively small number of mesogenic MA4 co-units, show that a slow site and a fast one are active for the reorientation of the guest tracer in the polymer matrix. This is indicated by the vertical dotted lines reported in Figure 3a.

All recorded lineshapes were carefully simulated. The distribution function of the spin probe sites was confirmed to be bimodal using the simulation procedure. In particular, the presence of a fast and a slow dynamic component provided the best simulation with respect to the ESR spectra [41] according to a two δ-like distribution function.

The ESR study on blends with the B20 or B10 copolymer as a minor component showed that the experimental lineshapes of blends were coincident at the same temperatures within experimental errors. Accordingly, the lineshapes and dynamic results presented in this study refer to both b5B10 and b5B20 blend samples.

**Figure 4:**
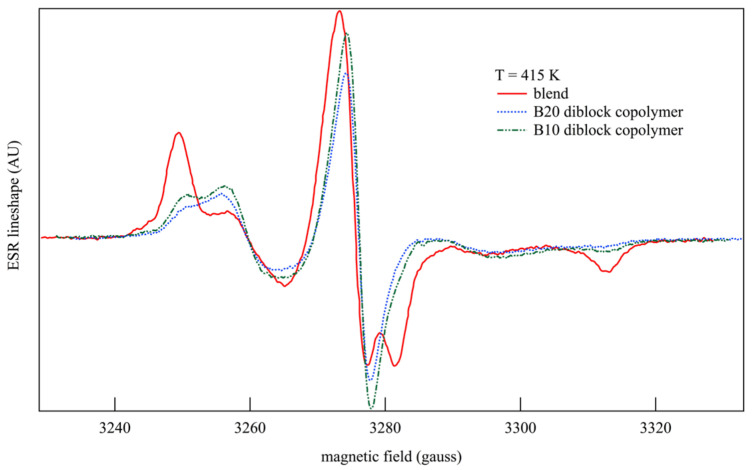
Comparison of the ESR lineshapes of B20 and B10 diblock copolymers and blends recorded at the same temperature of *T* = 415 K.

With respect to experiments on diblock copolymers recorded at the same temperature, the ESR lineshapes of blends turned out to be mostly dominated by the properties of the PMMA major component, with spectra shifted towards the slow-motion dynamics. This is observed by comparing the lineshapes of B20, B10, and blend samples that are recorded at the same temperature of *T* = 415 K (Figure 4). In fact, the peaks of the blend’s lineshape at the highest and the lowest values of the magnetic field are the uppermost ones with respect to the copolymers. Moreover, copolymers show that the external structures of their lineshapes shifted towards the center of the ESR spectrum, thus signaling that the molecular probe undergoes a faster rotational motion [90].

At temperatures less than 353 K, blend lineshapes were characterized by the ultra-slow-motion time scale, where ESR spectroscopy was insensitive to the details of molecular motion [57] (and references therein); then, the study of their rotational dynamics was investigated from temperatures that were greater than about 350 K.

Thus, it appears that ESR spectroscopy can reveal the features of dynamics at a time scale where local heterogeneity develops as a consequence of the polymer’s architecture and microstructure.

### 3.2. Rotational Dynamics

#### 3.2.1. Behavior with Temperature

**Figure 5:**
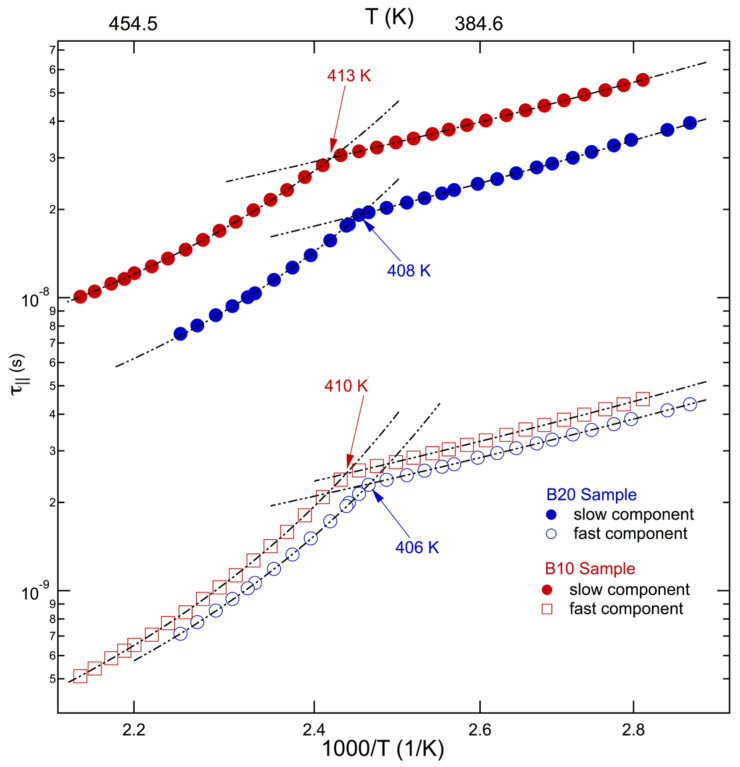
Temperature dependence of fast and slow rotational correlation times in B20 and B10 copolymers [55].

The investigation of the spinning correlation times of cholestane in diblock copolymers was carried out within a temperature range that exceeded *T_NI_*. These results were previously discussed in comparison with the dynamics of the random copolymers [55] of the same co-units. In this study, they are taken into account with the aim of establishing connections with the dynamics of their blends. In particular, the focus is devoted to relating analogies and differences to the different structures and architecture of materials at the nanometer length scale. The rotational dynamics results of copolymers are collectively shown in Figure 5. The presence of fast and slow sites for the probe’s reorientation indicates that the probe’s dynamics experience the presence of MA4 blocks independently of their concentration [52,55]. In particular, dynamic heterogeneity was attributed to the actual microphase separation of these diblock copolymers and the submicron/nanoscale domains formed via nematogenic MA4 blocks, which also occur at low concentrations. They favor the segregation of a portion of molecular tracers into the domains of the minority phase or provide probe redistributions among the block components above *T_ODT_*.

This analysis is supported by a comparison with the results of ESR dynamics in the random copolymers of the same co-units and comparable molar concentrations [52,55]. In these experiments, ESR evidenced a homogeneous reorientation instead as a consequence of the different environmental architectures at the nanometer scale.

As observed in Figure 5, the two copolymers show similar behavior, both in terms of shapes and magnitudes, with respect to the rotational correlation times as a function of temperature.

The investigated temperatures were greater than *T_g_*^PMA4^ and, for both slow and fast sites of the reorientation dynamics, two regions were identified. They are labeled as the intermediate (IT) and low (LT) temperature regimes (Table 4) in analogy with the nomenclature adopted in previous studies on random copolymers [55]. IT and LT dynamic regions are separated by a dynamic anomaly.

**Figure 6:**
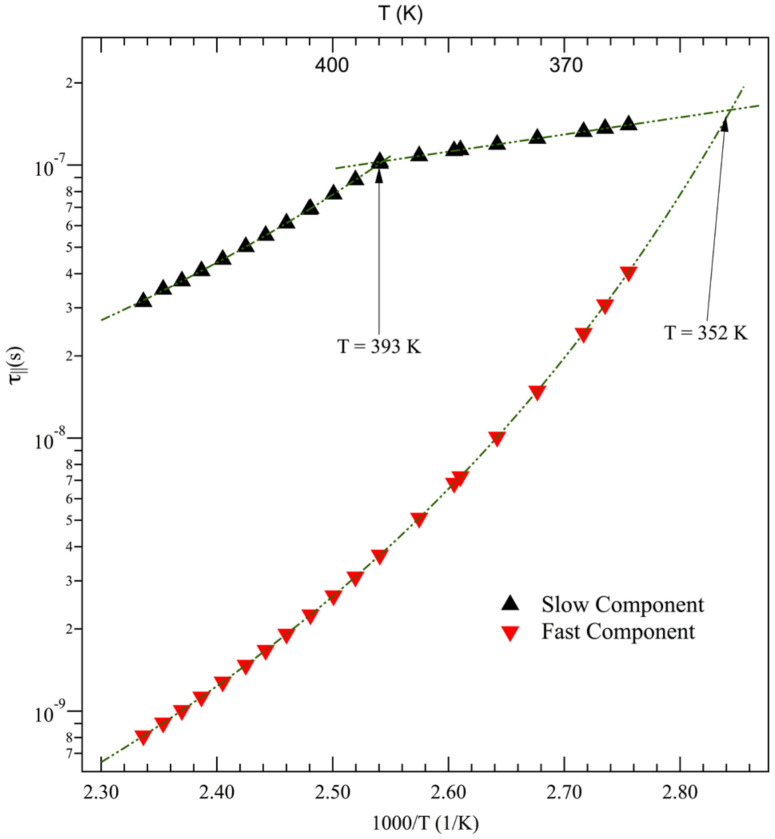
Arrhenius plot of rotational correlation times in blends.

The Arrhenius plot of ESR correlation times in the blends is reported in Figure 6. It accounts for their heterogeneous dynamics, with two sites available for the cholestane reorientation in the entire investigated range of temperatures. Note that the simulation of the blend’s lineshapes provided a tiny population of fast sites that are active in the entire investigated range of temperatures and that they are almost constant at a mean percentage of about 13%.

Figure 6 shows that, unlike diblock copolymers, fast and slow dynamic sites in the blends exhibit different behaviors in the entire investigated temperature range.

It appears that no dynamic anomaly was detected for the fast correlation times of the tracer’s rotation. Interestingly enough, an eye guide in the figure suggests a trend toward a collapse to homogeneous dynamics at the *T_NI_* temperature (Table 1), somewhat establishing a correlation of the fast site’s dynamics with the LC co-units of the minor component in the blend.

**Figure 7:**
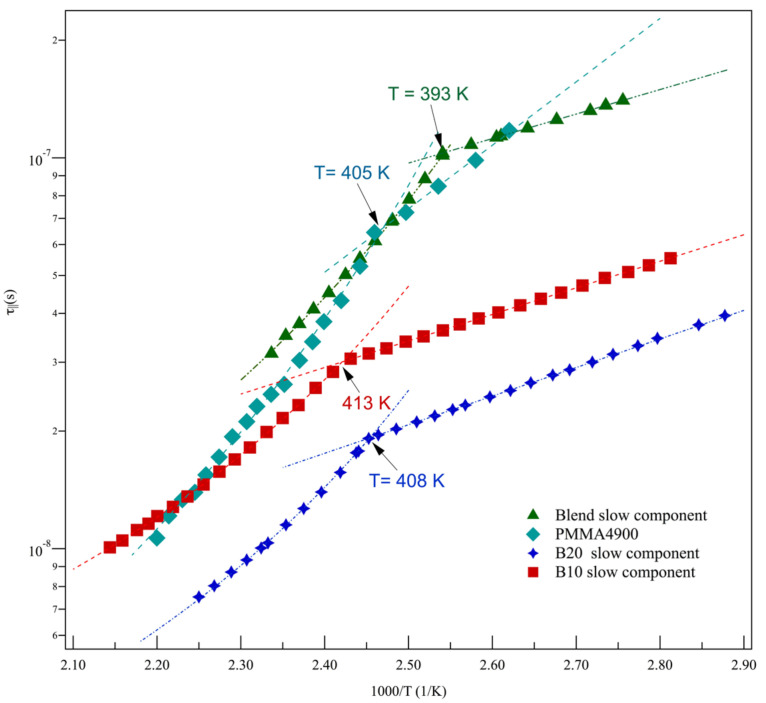
Temperature dependence of slow rotational correlation times in B10, B20, and blends. The temperature dependence of rotational correlation times in PMMA4900 [56] is also reported.

The slow sites present dynamic behavior that parallels the behavior of diblock copolymers; thus, a dynamic anomaly separates the IT and LT dynamic regions of the rotational correlation. As already noted in Figure 4, these correlation times are slower than the ones of diblock copolymers. A comparison with the ESR correlation times of the neat PMMA component of blends is not available. This is because PMMA lineshapes fall within the ESR ultraslow motion regime, where dynamical information is not accessed via spectroscopy. However, in Figure 7, the slow blend dynamics are confronted with the correlation times obtained via ESR experiments and they are carried out for the same temperatures in a PMMA4900 polymer [56]. With respect to the PMMA homopolymeric component of blends, the PMMA4900 sample had identical standard structure composition and lower mass.

The figure also shows, for comparison purposes, the slow dynamics of B10 and B20. It appears that the correlation times of PMMA4900 and blends fall within the same range of values if the same interval of temperature is considered. This finding suggests that the mechanisms of the relaxation of PMMA, as a major component of the blends, drive the slow correlation loss of molecular probes. On the other hand, the presence of a minor component in the blends makes the overall relaxation of the material faster and makes it possible to provide information on dynamics via ESR spectroscopy.

**Figure 8:**
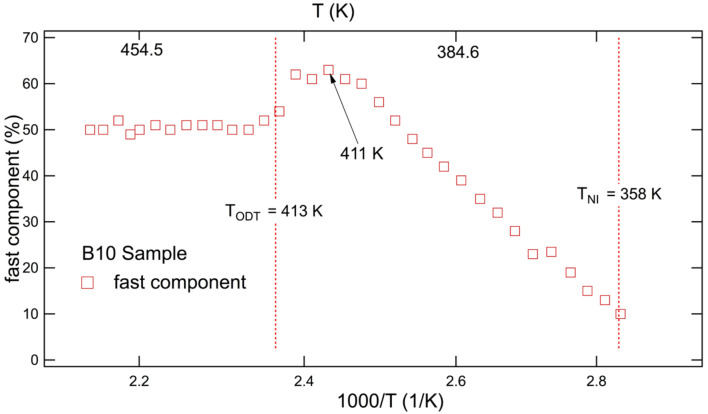
Population of the fast dynamic component in B10 [55].

As a final remark, the simulation of the heterogeneous ESR lineshape of diblock copolymers also provided the behavior of the population of dynamic sites over the temperature measurement range. In contrast to the blends, their values were not constant. An example is shown for B10 in Figure 8, where one can observe that different regimes can be detected. Well above both *T_ODT_* and *T_NI_*, fast sites show mean percentages that level at plateau values. It appears that the percentage of mesogenic co-units somewhat increases the fast population, which is increased in the presence of increased MA4 co-unit contents (60% for B20 and 50% for B10). With respect to lowered temperatures, at *T_ODT_* the percentage of fast sites increases. Recalling that an ordering process takes place in the nonmesogenic constituent of the block copolymers at *T_ODT_*, this result suggests that more free volume is made available for the mesogenic part of the copolymer at these temperatures where most molecular tracers are located [55]. Then, as temperatures further decrease towards *T_NI_*, the population of the fast dynamic component decreases progressively. This indicates that the increased tendency towards the nematic ordering in the LC block of the copolymer reduces the available free volume at the mesogenic units and pushes the molecular probe further away from it.

#### 3.2.2. Discussion

As a first step, let us consider the presence of the dynamic anomalies observed in Figure 5 and Figure 6, which separate the different temperature dependences of correlation times (Table 4). With respect to this, crossover temperatures between different dynamic regimes are not an unusual finding in complex systems. For example, they were also detected in ESR studies in the random copolymers of the same co-units [55] and at a temperature of about 1.2 *T_g_*^PMA4^, and they appear as a signature of different dynamical regimes. Moreover, studies have provided evidence [79,91,92] that the 1.2 *T_g_* temperature signals dynamic anomalies in molecular glass formers and linear homopolymers [56] (and the references therein). This is usually related to the onset of cooperative processes in materials.

Regarding blends, the dynamic anomaly in slow sites is observed at about the *T_g_*^PMMA^ temperature, as detected in B10 and B20 copolymers and the PMMA homopolymer (Table 1 and [59,93]). This indicates that the slow dynamics in blends are mainly driven by the coupling of the molecular probe to the *α* relaxation of the main chain, and this confirms what was argued in discussing the results of Figure 7.

In the case of diblock copolymers, the dynamic anomaly is located at about 1.2 *T_g_*^PMA4^ in the interval between the order-to-disorder transition temperature *T_ODT_* and *T_g_*^PMMA^ (Table 1). This finding suggests that a collection of different interactions can synergistically contribute to setting the crossover temperature of diblock copolymers. For example, one could consider the emergence, upon heating, of the collective processes of polymeric relaxation at temperatures above the PMMA glass transition of copolymers and the onset of cooperative processes related to the softening of the supramolecular structure and the nanodomains of the minority phase.

Crossover temperatures separate intermediate (IT) and low (LT) temperature regimes.

Below the crossover temperature, an activated Arrhenius regime is followed by the rotational relaxation (Table 4):

*τ_||_*(*T*) = *τ_||_* _∞_ exp [Δ*E*/(*k*_B_*T*)] (2)

The thermally activated regime, obeyed by the dynamics of the probe molecule, seems to denote that collective relaxation slowed down and could not drive the rotational diffusion of the molecular tracer. At this stage, coupling sets in with less cooperative and more closely localized relaxation mechanisms [94,95]. From the data reported in Table 4, the LT dynamics of slow sites in blends are affected and driven by the same relaxation processes that have been found to affect and drive the dynamics of both copolymer sites in the LT region.

The values of the activation energies, Δ*E*, are set at about 13 kJ mol^−1^ (Table 4) and the result is similar to the Δ*E* values found in the random copolymers [55] of the same co-units. Interestingly enough, Δ*E* shows the proper values of cholestane or of other molecular tracers dissolved in molecular glasses [96,97] or oligomers [57,98]. With respect to these, local segmental processes drive the activation regime of the “*cage*” where the probe is located, and this results in Δ*E* values within 10 kJ mol^−1^ to 22 kJ mol^−1^.

The activation energy of the present blends and their copolymers do not agree with the values reported for the low polymers of PMMA, which were investigated via ESR [56], or for other PMMA samples, which were investigated via dielectric or mechanical methods [94] (p. 258) [95]. However, a very interesting result comes from an ESR study carried out on a spin-labeled PMMA [99]. In that study, activation energies were detected with values comparable to the findings of this study [99] with respect to measurements carried out within the same temperature range.

Therefore, one may jointly consider the values of both the activated processes of a label—which is chemically attached to PMMA—and the random copolymers of the same co-units in which there is a small or absent fast population [55]. Accordingly, it can be argued that the localized mechanisms pertaining to the LT regions of relaxation have to be attributed to the cage’s stiffness and the constraints affecting the molecular tracers in the PMMA regions of copolymers and blends. Moreover, it can be noted that these relaxation mechanisms remain equally effective with respect to the fast and slow time scales of the probe dynamics of block copolymers. This can be recognized as a possible signature of the effects of the supramolecular order, constraining the phase-separated nanodomains of diblock copolymers.

A different dynamic regime is observed in the IT regions of copolymers and the slow sites of blends, as well as in the entire dynamic regime of the fast sites of blends. Let us consider first the copolymer case [55] as a starting point to better appreciate the different features that characterize the blend’s dynamics.

In diblock copolymers, as the temperature increases, softening followed by the vanishing of the ordered microstructure takes place. Therefore, at temperatures higher than *T_ODT_* > *T_g_*^PMMA^ the relaxation dynamics become somewhat sensitive to the less localized collective dynamic processes of the polymeric material. Then, the VFT (Equation (3)) temperature dependence of correlation times is observed:

*τ_||_*(*T*) = *τ_||_* _∞_ exp [*T_b_*/*T* − *T*_0_] (3)

Table 4 reports dynamic VFT regions, pseudo-activation temperatures *T_b_*, and Vogel temperatures *T*_0_.

In the eventual virtual coincidence of *T*_0_ pertaining to the VFT fitting of ESR and viscosity measurements, it is possible to evaluate the degree of coupling between rotation and viscosity—namely, between macroviscosity and microviscosity—at the level of segmental friction. This is carried out by relating spinning correlation times and viscosity via a power law:

*τ_||_*(*T*) ∝ [*η* (*T*)]*^ξ^* (4)

In Equation (4), *ξ* is a fractional exponent and may range between 0 and 1. In the case of *ξ* = 1, a complete coupling of the probe dynamics to the terminal relaxation of the host matrix holds. *ξ* is the ratio of the pseudo-activation temperature *T_b_* of the VFT law pertinent to the rotational dynamics over the *T_b_* value of the viscosity of the sample.

In a polymeric matrix, the occurrence of complete coupling between structural relaxation processes, or viscosity, and reorientational spin probe dynamics was demonstrated in very few cases [53,56,57]. In the IT dynamic range and the presence of liquid crystalline units, an exponent *ξ* lesser than unity and the consequent decoupling was interpreted as the outcome of the coexistence of steric and cooperative effects [41,55].

In the present case, B10 and B20 diblock copolymers show thermorheological complexity [100,101,102], and viscosity *η* and shift factor *a* (*T*) may not present coincident *T*_0_ values of the two different material functions (Table 2).

Comparing *T*_0_ temperatures from ESR (Table 4) and the ones from rheology (Table 2), it is observed that both dynamic sites of B10 follow a VTF where the *T*_0_ temperatures relative to molecular reorientation agree with the Vogel temperatures of shift factor *a* (*T*) [55]. This finding indicates that the structural relaxation or very low index modes, which pertain to the dynamics of the chain at long times, are not effective with respect to rotational relaxation, while modes generated by the subunits of the chain itself take on relevance.

A rescaling procedure with respect to these relaxation mechanisms may be carried out according to

*τ_||_*(*T*) ∝ [*a* (*T*)]*^ξ^* (5)

and the values of exponent *ξ* of the power law are reported in Table 4.

It was demonstrated [55,56] that, in the IT region, the decoupling degree of the rotational dynamics of cholestane tracers with respect to viscosity should be ascribed to both the presence of cooperative processes and steric hindrance effects in polymers. Steric hindrance, for example, might be related to the connectivity of the polymer or the presence of side groups, mediating the interaction between chain dynamics and tracer diffusion. Here, a comparison between the decoupling parameter of the rotational relaxation obtained for both copolymers with respect to the respective shift factors could be carried out since the steric hindrance of both copolymers refers to the presence of the blocks of the same co-units.

The *ξ* values of the fast and slow components of B10 and B20 diblock copolymers are comparable and slightly diminish as the percentage of MA4 units increases. The thermoreologically simple random copolymers of the same co-units were investigated in a previous study [55]. With respect to diblock copolymers, their fast sites in IT regions exhibited a similar *ξ* dependence on MA4 percentage; in this case, however, a greater coupling of fast dynamics was observed with respect to the slow one. On the other hand, diblock copolymers show comparable coupling over the two time scales of molecular relaxation. According to the discussion of their activation energies, this outcome could also be interpreted as a marker, for block copolymers, of their tendency to form ordered supramolecular structures.

Regarding the blends, the VTF parameters that characterize their dynamic responses in the proper intervals of temperatures are also shown in Table 4. At first glance, slow blend sites recall the trend of copolymers. A more in-depth analysis of the data shows that the dynamic anomaly is set within the range of the *T_g_*^PMMA^ values of neat PMMA samples. Moreover, the *T*_0_ temperature of the slow VLF site of blends agrees with the trend suggested by the ESR study on PMMA samples at lower masses [56] and with the one obtained via viscosity measurements [59]. They were observed at around 285 K. This provides a nice support to the discussion of Figure 7, ascribing the coupling of the slow relaxation of blends to the *α* relaxation of the neat PMMA main chain.

It has already been noted that the fast relaxation of blends shows a unique dynamic trend without any dynamic anomalies. We can describe this as a VTF behavior that starts above the *T_NI_* of PMA4 co-units, as detected in the neat diblock copolymers, and then passes smoothly through the *T_g_*^PMMA^ and extends up to about 430 K. This indicates that, on this fast time scale, the rotational correlation tracks segmental relaxation modes that are not yet frozen below *T_g_*^PMMA^. The Vogel temperature *T*_0_ in Table 4 allows relating the relaxation of fast sites to the relaxation driven by large subunits of PMMA, as indicated by the *T*_0_ value of the fast collective relaxation. Nevertheless, this fast relaxation is strongly connected to the presence of an LC block, aiming to acquire an ordered texture at *T_NI_*, as suggested by the ideal merging of slow and fast dynamics at *T_NI_* (Figure 6).

With this respect, it has to be noted that ESR investigations have already manifested the ability to detect similar peculiarities of dynamics. Indeed, they occur at nanometer length scales and on a time scale where local heterogeneity, concentrations, and self-concentration fluctuations interplay with one another as a consequence of the polymer architecture or its dynamics. A study carried out on a random copolymer of MMA-MA4 (40% MA4) co-units revealed the presence of a *T_NI_* temperature of the polymer after extrapolating the curve of fast sites, however, the nematic–isotropic phase was not detected macroscopically [98]. The analysis of this result, together with the results on random mesogenic copolymers comprising the same co-units [55] (and discussions therein) and the results of B10/B20 diblock copolymers, allowed locating the fast sites at the mesogenic groups/blocks of the polymers with an amount that is dependent on the percentage of MA4 units in the copolymers.

After collecting all this information, a possible identification of the site, where these fast relaxation mechanisms in the blends are active, is found in the few available regions of MA4. This is also corroborated by the tiny population of fast sites in blends.

More dynamic insight into the overall rotational relaxation in blends might be provided by considering a scaling procedure of correlation times with respect to macroscopic relaxation properties. Due to the lack of viscosity measurements on the blend samples, we considered the possibility of evaluating their coupling degree with respect to the PMMA homopolymer, which has been demonstrated to drive their rotational relaxation at the nanoscale. To this aim, a PMMA sample from the literature [59] (referred to as PMMA22R in Table 2) was taken into account. Its tacticity and molecular weight were comparable to that used as the major component in the blends. In particular, this specific PMMA sample was selected because of the virtual coincidence of the *T*_0_ values with respect to its rheological behavior (Table 2) relative to the behavior of blends in ESR dynamics (Table 4). In this manner, a rescaling procedure was allowed, providing the calculation of the *ξ* exponent with respect to viscosity according to Equation (4).

For slow sites, the *ξ* exponent denotes poor coupling to the main polymer’s relaxation. The fractional exponent of the slow dynamics of blends is comparable to the *ξ* plateau value found in the IT region within the framework of a study on a PMMA series [56]. It is also comparable to the fractional exponent pertinent to the IT region of the dynamic rotation of the random MA4-MMA copolymer R10, containing only 10% of MA4 co-units [55]. In both cases, the samples exhibited homogeneous dynamics, with only one site found for the probe’s rotation. Therefore, the present value for *ξ* would confirm that *α* relaxation processes, available in neat PMMA polymer and the amorphous regions of the diblock polymers of blends, would mainly affect the dynamic behavior of slow sites.

Concerning fast dynamics, greater values were observed for the *ξ* exponent. This behavior parallels the greater coupling exhibited by fast sites in MA4 homopolymers [41], in random copolymers of the same MA4-MMA co-units with high liquid crystallinity [55,98], or the one found in a closely related nematic polyacrylate [82].

In agreement with these results [55,98], a further effective indication is obtained with respect to the location of blends for the probe undergoing the fast reorientation, which is observed in the mesogenic side groups of the diblock co-unit.

As a final remark, it appears that the adopted rescaling procedure of fast dynamics in blends with respect to the VTF behavior of PMMA [59] provides consistent interpretative support for dynamic behavior at the nanometer length scale and for the nanosecond times of blends.

## 4. Conclusions

Due to the sensitivity of ESR spectroscopy, the present study provided a valuable scenario with respect to the dynamics of the blends of diblock copolymers at nanometer and nanosecond scales.

Fast and slow molecular sites with different relative populations over large temperature ranges were detected for the reorientation of the cholestane molecular tracer as a consequence of the heterogeneous morphology and dynamics of these materials at the nanoscale. In particular, the persistent presence of fast sites provided the signature of phenomenon of microphase separation and/or confinement in submicrometer/nanometer domains; this is shown to be effective in blends, even at low MA4 co-unit contents in the copolymer partners. This peculiarity was also previously observed in diblock polymers.

The self-assembling ability in the supramolecular structures of diblock copolymers led to almost parallel trends for the temperature dependence of fast and slow correlation times, with a dynamic crossover that is observed also in the literature for polymers and glass formers at the onset of cooperative processes and located at 1.2 *T_g_*. The crossover was detected at a temperature of about 1.2 *T_g_*^PMA4^, however within a dynamic interval where *T_ODT_* and *T_g_*^PMMA^ also provide contributions synergistically.

In blends, the fast and slow sites of rotational relaxation characteristics seemed to be selectively connected to their different morphological properties. The temperature behavior of the slow sites dynamics was similar to the one of diblock copolymers. Nevertheless, the dynamic change was found at *T_g_*^PMMA^; an α-β relaxation splitting was mostly recalled, which was driven by the homopolymer PMMA, as the major component of blends. Fast sites, which were free from dynamic anomalies in the investigated temperature range, suggested the presence of a merging at *T_NI_*. Their presence was related to molecular probes that were located at the LC block of the copolymer component of the blends.

More insight was provided by the evaluation of the coupling degrees of the rotational correlation times relative to the viscosity or structural relaxation dynamics, shining a light on the mechanisms of relaxation. In particular, in blends, cooperativity was related to the properties of the polymer chain of the majority component, which also determined the overall relaxation trend of fast site dynamics. This is different from the results on rotational dynamics in block copolymers, which appeared to be driven by and coupled to more local relaxation processes due to the monomeric friction coefficients of the two blocks and the thermorheological complexity of the resultant polymer.

Lastly, a convenient characterization and the location of the sites available for the molecular reorientation were provided via the analysis of coupling data and of dynamic signatures.

## Figures and Tables

**Table 1:** Physical and chemical characterization of copolymers and blends.

Sample	*x* (mol%)	*M_n_* (kg mol^–1^)	*M_w_*/*M_n_*	*T_g_*^PMA4^ (K)	*T_g_*^PMMA^ (K)	*T_NI_* (K)	*T_ODT_* (K)
PMMA	0	21	1.27	---	385	---	---
B20	20	34	1.31	324	394	358	413
B10	10	27	1.27	328	397	353	423
b5B20	20	---	---	---	386 ^2^	---	---
b5B10	10	---	---	---	386 ^2^	---	---
PMA4	100	29.9	2.43	305	---	357	---
PMMA22R ^1^	0	24	1.04	---	392	---	---

^1^ Data of sample PMMA22R are reported from the literature [59]. PMMA22R presents the same standard microstructure [60] as the PMMA polymer of this study. ^2^ Calculated according to the Utracki and Jukes equation [61].

**Table 2:** VFT parameters of B20 and B10 copolymers.

Sample	Variable	*η*_∞_ (Pa s)	*T_b_* (K)	*T*_0_ (K)
B20	*η* (*T*)	(3.00 ± 0.05)10^2^	210 ± 30	330 ± 30
	*a* (*T*)	-	1450 ± 50	340 ± 4
B10	*η* (*T*)	(7.24 ± 0.05)10^−2^	1800 ± 250	240 ± 20
	*a* (*T*)	-	1950 ± 50	323 ± 3
PMMA22R ^1^	*η* (*T*)	1.5 ± 0.5 10^−5^	3530 ± 50	286 ± 3

^1^ Data of the PMMA22R sample from the literature [59].

**Table 3:** Components of the magnetic tensors expressed in the molecular reference frame.

Samples	*g_xx_*	*g_yy_*	*g_zz_*	*a_xx_* (Gauss)	*a_yy_* (Gauss)	*a_zz_* (Gauss)
PMMA4900 ^1^	2.0026	2.0093	2.0066	32.3	6.0	5.0
HomoPMA4 ^2^	2.0026	2.0092	2.0069	32.6	5.5	5.0
B20 ^3^, B10 ^3^	2.0026	2.0092	2.0069	32.9	5.5	5.0
Blends	2.0026	2.0092	2.0069	32.9	5.5	5.0

Data from: ^1^ [56], ^2^ [41], ^3^ [55].

**Table 4:** Temperature regions and the dynamic parameters of the block copolymers and blends.

Sample	*T* Range (K)	*T* Region ^1^	Law ^2^	*τ*_||∞_ (s)	*T*_0_ (K)	*T_b_* (K)	*ξ* ^3^	Δ*E* (kJ mol^–1^)
B20	445–406	IT (F)	VFT	(2.5 ± 0.2) × 10^−11^	340 ± 7	200 ± 20	0.14 ± 0.01	---
	406–347	LT (F)	Arr	(5.5 ± 0.2) × 10^−11^	---	---	---	14 ± 1
	445–408	IT (S)	VFT	(4.7 ± 0.2) × 10^−10^	340 ± 7	180 ± 25	0.12 ± 0.02	---
	408–347	LT (S)	Arr	(3.1 ± 0.2) × 10^−10^	---	---	---	15 ± 1
B10	467–410	IT (F)	VFT	(2.2 ± 0.2) × 10^−11^	323 ± 7	350 ± 30	0.18 ± 0.02	---
	410–357	LT (F)	Arr	(5.5 ± 0.2) × 10^−11^	---	---	---	13 ± 1
	467–413	IT (S)	VFT	(9.8 ± 0.2) × 10^−10^	323 ± 7	260 ± 25	0.13 ± 0.01	---
	413–357	LT (S)	Arr	(6.9 ± 0.2) × 10^−10^	---	---	---	13 ± 1
b5B20 and b5B10	428–363	(F)	VFT	(4.3 ± 0.3) × 10^−12^	276 ± 5	795 ± 20	0.23 ± 0.01	---
	428–393	IT (S)	VFT	(6.0 ± 0.2) × 10^−10^	276 ± 6	604 ± 20	0.17 ± 0.01	---
	393–363	LT (S)	Arr	(2.6 ± 0.2) × 10^−9^	---	---	---	12 ± 1

^1^ Key: Low-temperature (LT) and intermediate-temperature (IT) region. The slow (S) or fast (F) index specifies the dynamic component. ^2^ VFT (Equation (2)) or Arrhenius behavior. ^3^ The *ξ* coefficient for blends was calculated with respect to the *T_b_* value of the PMMA of comparable molar mass from the literature (sample PMMA22R in ref. [59]).

## Data Availability

The data presented in this study are available in the present article.
